# “Your heart keeps bleeding”: lived experiences of parents with a perinatal death in Northern Uganda

**DOI:** 10.1186/s12884-022-04788-8

**Published:** 2022-06-15

**Authors:** Anna Agnes Ojok Arach, Juliet Kiguli, Victoria Nankabirwa, Noeline Nakasujja, David Mukunya, Milton W. Musaba, Agnes Napyo, James K. Tumwine, Grace Ndeezi, Joseph Rujumba

**Affiliations:** 1 Department of Nursing and Midwifery, Faculty of Health Sciences, Lira University, Lira, Uganda; 2 Department of Paediatrics and Child Health, School of Medicine, Makerere University College of Health Sciences, Kampala, Uganda; 3 Department of Community Health and Behavioural Sciences, School of Public Health, Makerere University College of Health Sciences, Kampala, Uganda; 4 Department of Epidemiology and Biostatistics, School of Public Health, Makerere University College of Health Sciences, Kampala, Uganda; 5 Centre for Intervention Science and Maternal Child Health (CISMAC), Centre for International Health, University of Bergen, Bergen, Norway; 6 Department of Psychiatry, School of Medicine, Makerere University College of Health Sciences, Kampala, Uganda; 7 Department of Community and Public Health, Busitema University Faculty of Health Sciences, Mbale, Uganda; 8 Department of Obstetrics and Gynaecology, Busitema University Faculty of Health Sciences, Mbale, Uganda

**Keywords:** Lived experiences, Experiences, Stillbirths, Early neonatal deaths, Parents, Men, Women, Perinatal death, Uganda

## Abstract

**Background:**

Worldwide, two million babies are stillborn and 1.8 million babies die before completing seven days of life. Approximately 4% of pregnant women in Uganda experience perinatal death. The response following a perinatal death tends to be socio-culturally constructed. Investigating the unique personal experiences of parents from a low-income setting with unique cultural beliefs and practices is crucial for the design and implementation of appropriate interventions.

**Objective:**

To describe the lived experiences of parents following perinatal death in Lira district, Northern Uganda.

**Methods:**

A qualitative study was carried out drawing on the tenets of descriptive phenomenology. We conducted 32 in-depth interviews in Lira district, Northern Uganda between August 2019 and September 2020 with 18 women and 14 men who had experienced a stillbirth or an early neonatal death within the preceding 2 years. Participants were selected from different families and interviewed. A local IRB approved the study. All in-depth interviews were audio-recorded, transcribed, translated, and data were analysed using a content thematic approach. Key findings were discussed based on Worden’s Four Tasks of Mourning theory.

**Results:**

The themes that emerged from the analyses included reaction to the perinatal loss and suggestions for support. The participants’ immediate reactions were pain, confusion, and feelings of guilt which were aggravated by the unsupportive behaviour of health care providers. Men cumulatively lost financial resources in addition to facing multiple stressful roles. Delayed reactions such as pain and worries were triggered by the sight of similar-age-babies, subsequent pregnancy losses, and marital challenges. Participants recommended emotional support and management of postnatal complications for parents faced with perinatal loss.

**Conclusion:**

Losing a baby during the perinatal period in a resource-constrained setting negatively affected both gender. In addition, men suffered the loss of financial resources and the burden of multiple stressful roles. Acknowledging the pain and offering support to the grieving parents reinforce their coping with a perinatal loss. In addition to family and community members, health care providers need to provide emotional support and postnatal care to parents who experience perinatal death.

## Background

Worldwide, 2 million babies are stillborn and 1.8 million die before completing 7 days as reported in 2019 [1, 2]. Almost all of these deaths (98%) occur in low- and middle-income countries (LMIC), with sub-Saharan Africa and South Asia being the most affected [2]. Perinatal death disproportionately affects population that are socially and economically disadvantaged. For instance, in 2019 the stillbirth risk in sub-Saharan Africa was about 21.7/1000 total births compared to 2.9/1000 total births in Western Europe [2]. The estimated stillbirth risk in Uganda is similar to Kenya and Tanzania at about 17.8/1000 total births [2]. These deaths have been associated with risk factors such as maternal age, infections, nutrition and lifestyle factors, and socioeconomic factors [3–8]. Rural areas are reported to contribute higher proportions of perinatal deaths than peri/urban areas [5, 7].

Perinatal death can negatively affect women and men not just physically and emotionally [9] but also in terms of long-lasting economic and psychosocial wellbeing [10]. Most family members experience the effects of perinatal death individually [11]. Although parents experience painful grief after a perinatal loss [12], the grief reactions are shown to vary across gender [13]. Some studies have noted that the grief reactions are aggravated by insensitive health systems, health care providers, friends, a strained marital relationship and financial burdens [14–17]. Although most of these publications are from high- and middle-income countries, there is limited evidence from low-income countries. Furthermore, men as parents to stillborn babies or early neonatal deaths have received less attention in terms of research. Even though studies from other contexts show that after a perinatal loss, men tend to hide their grief, provide support to their female partners and get involved in decision making and practical tasks [18–22], little is known about their experience after perinatal death. Moreover, grief reactions of persons with a perinatal death are said to be influenced by the social and cultural context [23, 24]. There are a limited number of publications that have documented the experience of Ugandan parents who experienced perinatal deaths [16, 25]. Given the several ethnic groups in Uganda with varying cultural beliefs and practices which may have a bearing on how perinatal loss is experienced in different contexts. Northern Uganda is poorly studied despite the high perinatal mortality rate (~ 40/1000 births) [3].

Several authors assert that persons with a perinatal death, if not attended to, may experience disorders such as anxiety, complicated grief, depression and posttraumatic stress disorders [26–30]. These complications are reported to have an adverse outcome that may extend into the next pregnancy or may become prolonged [31–34] resulting in a disorganized mother-infant attachment, marital break-up and financial hardship [10, 11, 35, 36]. Investigating the unique personal experiences of perinatal death is crucial, for developing culturally appropriate interventions to provide care and support to reduce the impact. The aim of this study was to describe the lived experiences of parents following perinatal death in Lira district, Northern Uganda. The lived experience was defined as a personal account of the events gone through after experiencing a perinatal death. Worden’s Tasks-based Theory of Mourning [37] was used in understanding how parents processed their responses to perinatal deaths. The theory presents that after the loss of a loved one, one is normally faced with a number of tasks to accomplish as they try to adapt to the loss. The adaptation strategies include, Task I: accepting the reality of the loss which involves facing the reality that the person is dead and will not return which involves both intellectual and emotional acceptance. Activities such as funeral rites and counselling help one to adjust to the loss. Task II: processing the pain of grief which involves experiencing the emotions and sensations among others as part of the mourning process. Task III: adjusting to a world without the deceased which include external, internal and spiritual adjustment, and Task IV: finding an enduring connection with the deceased in the midst of embarking on a new life. This 4th task involves emotionally keeping the dead person in one’s life as one continues with life. This theory was chosen to describe the lived experiences after perinatal death because it views bereavement as a process of adapting to the loss (death), the tasks are not linear and suits the unique mourning processes of individuals. In addition, the theory provides for the integration of the socio-cultural beliefs and practices of the study community in understanding the lived experiences.

## Methods

### Study setting

The study was carried out in Lira, a district in Northern Uganda where the Survival Pluss trial (NCT0260505369), a cluster randomized community-based trial had taken place. The detailed description of the trial has been published elsewhere [38]. Lira district had a population of 410,000 in 2014 [39], served by 31 healthcare facilities, including one referral hospital, 3 healthcare centres with surgical theatres, 17 healthcare centres with maternity wards but no surgical facilities, and 10 healthcare centres (dispensary). Subsistence farming is the main economic activity in the region. The dominant population are the Lango who speak the *Lango* language. The Lango culture is predominantly patriarchal and men are more valued and respected in decision making than women [40]. Women are most commonly excluded in decision making regarding family issues, for instance, pregnancy and childbirth. In this society, emphasis is put on bearing children for the preservation of lineage and having at least a male child is most preferred [41]. The study was carried out in 3 out of 13 sub-counties, Aromo, Agweng and Ogur. These sub-counties formed the study site for the Survival Pluss trial. Located in the hard-to-reach areas of the district, the sub-counties were chosen based on reported poor maternal and perinatal indicators.

### Study design

This qualitative study was conducted as part of mixed-method research and draws on the principles of descriptive phenomenology design. Phenomenology is a philosophy and research method of gaining insight into the lived experiences of a phenomenon as described by persons who have lived it [42] in this case a perinatal death. Descriptive phenomenology was founded by Edmund Husserl (1859–1939), a German philosopher. According to Husserl (1970), the meaning of lived experiences can be described through an interaction between the researcher and the objects being studied during interview and observation [43]. A researcher has to bracket previous understanding or misconception of the phenomena to allow new meaning to emerge. This design was chosen because it gives a rich description of parents’ lived experiences in relation to losing an infant they were attached to from conception until birth. It allows participants to construct their interpretation and understanding of the infant loss.

### Study population

Participants were women and partners of women who had had either a stillbirth or an early neonatal death within the past 2 years. Those who lived in the study area (Aromo, Agweng and Ogur sub counties) from at least the third trimester (≥ 28 weeks of gestation) until 6 months after perinatal death were included in the study. Their participation was irrespective of whether perinatal death occurred in a health facility or at home. The duration of staying in the area was to allow the participant’s experiences to emerge within the study context. Participants were excluded if they had migrated to distant places beyond the reach of the study team or were not willing to talk about the perinatal deaths. The 2 years duration from the date of a perinatal death to the time of the interview was considered appropriate to interact with participants when grieving had already taken place. Previous studies suggest that normal grieving after a perinatal loss declines over a period of 2 years [31, 44]. Participants in a previous study preferred research contact between 1 to 2 years, though 6 months from the time of loss was also considered appropriate [45]. Others report that, after 6 months with no improvement, the normal grieving may become prolonged [46]. Participants who experienced a perinatal death during the Survival Pluss trial were purposively selected from the trial records. The trial record had addresses of the participants who experienced a perinatal death, date of childbirth and death of the neonates. Top up was made with women who had not participated in the Survival Pluss trial. They were identified as next of kin to perinatal deaths from the health facility registers or through interaction with community volunteers and leaders and traced with the help of the community leaders. Similarly, men whose partners had experienced a stillbirth or an early neonatal death were identified in the same way as the women. A study team member accompanied by a community volunteer or a leader visited the homes of the potential participants to seek permission to participate in the study. For married women, the partners had to permit their participation. A total of 61 potential participants were identified: 27 from Survival Pluss trial records, 11 through health facilities records, and 23 through interaction with community leaders. Out of the 61, 32 were interviewed (18 women and 14 men).

### Data collection

Data were collected using semi-structured in-depth interviews (IDIs) between August 2019 and September 2020. In-depth interviews were preferred because of the sensitivity of the information collected from the participants. The researcher and an assistant who are fluent in the local language *Lango* collected the data in a face-to-face interview at the participant’s home. Before each interview, the research assistant visited the participant at his/her home and confirmed participation as well as the time and place they found convenient. During the interview, non-verbal expressions were observed and recorded while verbal discussions were audio-recorded using a mobile phone. Verbal discussions were audio-recorded for transcription later and also to give ample time for participant-interviewer interactions. Each interview took about 60 to 90 minutes. Data collection with women was completed before interviewing men. For each group, data were collected until issues being discussed got repeated implying saturation of the responses. These were observed during the 12th participant and a few more participants were added to complete the data collection. The interviews with women were facilitated by a female (first author) and men were interviewed by a male research assistant. This is because men felt more comfortable discussing their issues with a male interviewer. The data collection tool was translated and pretested. It had questions on participant demographics, labour and delivery experience of women/spouse, events surrounding the death, how they felt immediately death was confirmed and the support they desired or received.

### Research team and reflexivity

The research team consisted of the three authors AAAO, JK, JM and two research assistants. The authors conceived the study. The first author who is a nurse specialising in maternal and child health originates from the study region. She was always present during data collection and also trained the research assistants. She and the male research assistant had previously worked with the study community during the Survival Pluss trial and therefore, they were familiar to some of the participants. The data collection team always had a thorough introduction with the participant families to establish rapport and trust. In her past, this author witnessed close friends suffer from stillbirth after normal first pregnancies. These and those she witnessed during her practice in the maternity setting inspired her to understand the experience of women and men who underwent a perinatal loss. The transcripts were prepared by one of the authors (a nurse) who collected the data and was assisted by the research assistants. One author conducted the analysis and was verified by two other authors. The two authors are senior academic researchers with background in sociology, anthropology and extensive experience in qualitative research.

### Analysis

Audio records and field notes were transcribed in the local language *Lango* and the transcripts were verified with the audio record/field notes. *Lango* transcripts were then translated by an experienced translator and professional *Lango* language teacher (MA in Applied African Linguistics). The study team verified translated transcripts to ensure the meanings were maintained. Before analysis, each transcript was read exhaustively to familiarize with the data. Data was saved on the researcher’s computer and encrypted. A copy accessible by the researcher only was backed up in the cloud. Data were analysed using a content thematic approach [47, 48] with the help of NVivo software [49]. Each transcript was imported into NVivo. According to Elo [47], qualitative content analysis is aimed at the attainment of a condensed and broad description of the phenomenon. Thematic analysis helps in identifying patterns of meaning across the whole dataset [48]. Codes were generated based on significant information from the transcripts. Some codes were developed a priori from previous studies. Similar codes were grouped into sub-themes. The themes were created from the sub-themes. Verification of the codes, sub-themes and themes were performed by the research team members. Appropriate participants’ expressions were identified and included as quotes to illuminate the data. This study is hinged on Worden’s Four Tasks of mourning Model [37] to explain the different themes and sub-themes that emerged.

## Results

**Fig. 1:**
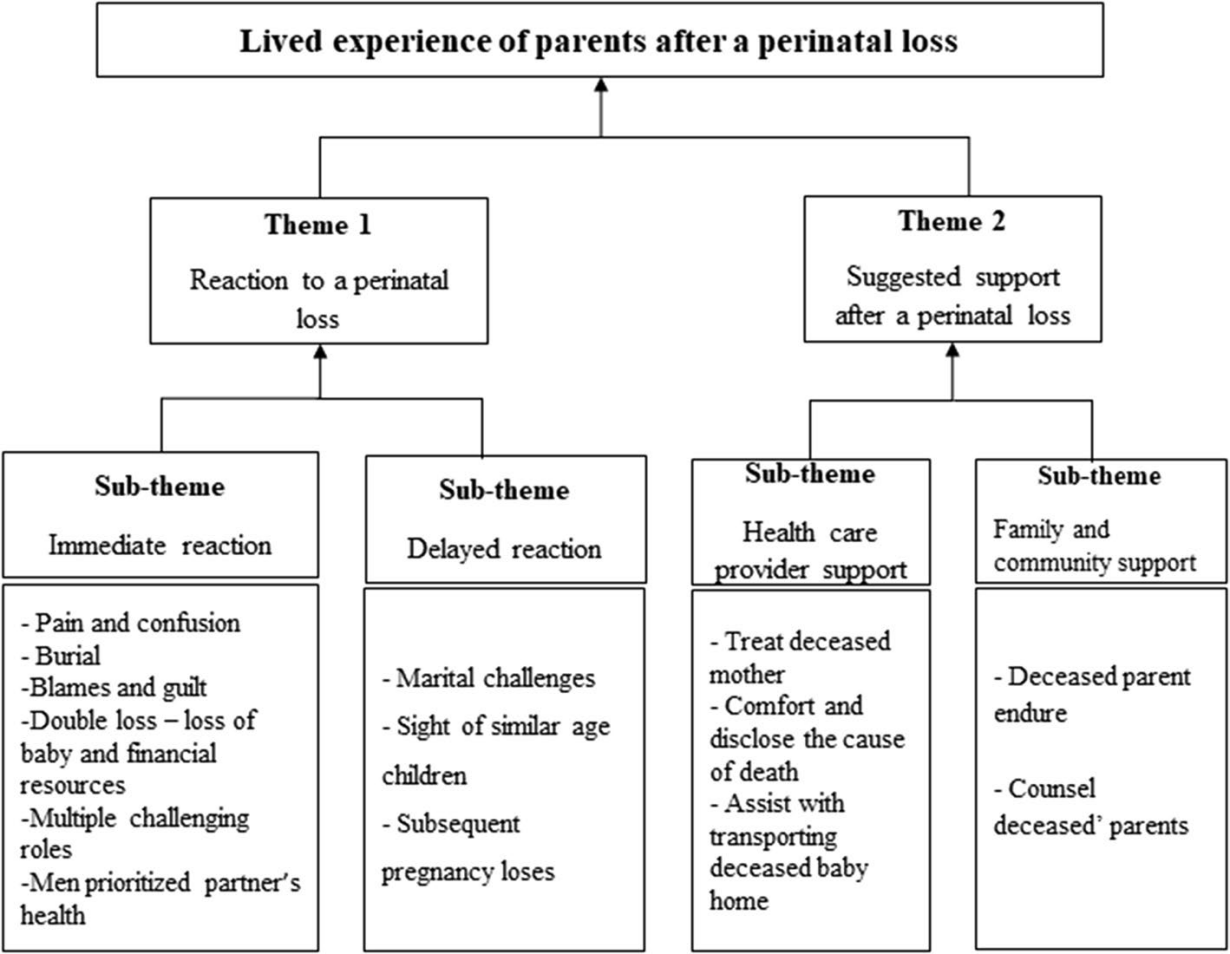
Themes and sub-themes

**Fig. 2:**
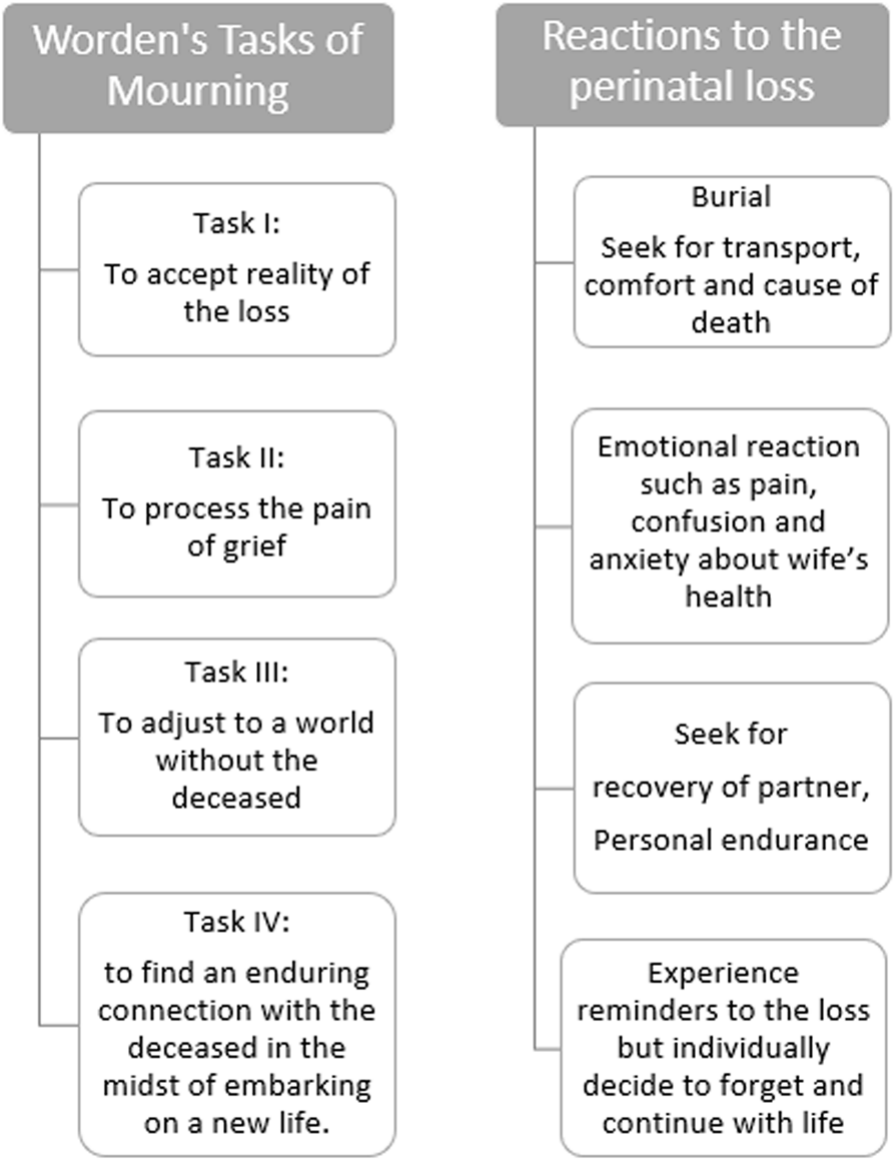
Reaction to the perinatal loss presented according to Worden’s Tasks of Mourning theory

Thirty-two (32) participants consisting of 18 women and 14 men with the age ranging from 17 to 68 years were interviewed about their experience following a perinatal death. Almost all were married (93.8%) and subsistence farmers (90.6%). The characteristics of the participants are found in Table 1. The following themes emerged from the analysis: reaction to a perinatal loss and suggested support after a loss. The reactions of the participants following perinatal deaths were immediate or delayed. They suggested bereavement counselling and postpartum care for parents who experienced a perinatal death. The themes are explained in detail and also presented diagrammatically in Figs. 1 and 2.

**Table 1:** Characteristics of participants interviewed about their experiences following perinatal death in Lira, Uganda 2019–2020

Characteristics	Women	Men
	***N*** = 18	***N*** = 14
Age		
≤ 19	2	1
20–30 years	10	9
> 30 years	6	4
Level of Education		
No formal education	5	0
Primary	12	7
Secondary or higher	1	7
Marital status		
Married	16	14
Single	2	0
Parity		
Primipara	5	5
Para 1–4	6	5
Multipara	7	4
Main occupation		
Subsistence farmer	18	11
Teacher	0	4
Type of child death		
Stillbirth	14	7
Early neonatal death	4	7
Place of child death		
Home	8	5
Health facility	10	9

### Theme 1: reaction to a perinatal loss

When the participants heard about the perinatal death, they greatly felt the impact and reacted in different ways. There were immediate and delayed reactions. In terms of grief, the reaction was similar to stillbirth and an early neonatal death.

### Sub-theme 1: immediate reaction

The immediate response reported by participants after perinatal loss were pain, confusion, facing multiple challenging roles, concern about the health of the partners, health care providers’ reaction, blame and guilt. These are described below:

#### Pain

Participants reported that losing their children was associated with lots of pain. They narrated feeling pain, heartbroken, torn apart, hurt and hard life because they returned home with no baby. Participants felt they had lost the future benefits associated with the children. For instance, a boy would become the heir and support the family or a girl would be married off and bring wealth and social prestige to the family. Some of the study participants narrated how they had endured complicated pregnancies. A participant acknowledged that her baby comes directly from her body, and that makes it very painful. The pain was reported as severe for those who were having consecutive losses or index experience. Men reported much pain especially when it was a boy child or the firstborn. They mourned but not openly. Participants who remembered the most painful experience were emotional during the interview. The pain could be because they had permanently lost a precious gift as known in the study setting.

*“I felt heartbroken because, on her side, this was a male child. Like we lived, we never had any boy so we were happy to have got one and I had high hopes for it. I really became weak upon seeing the dead baby.”* P 2003, A 41-year-old polygamous father of 9.

#### Confused

More than half of the participants felt confused on hearing that the outcome of their pregnancy was a perinatal death. They reported being confused, ‘lost in thoughts’, stressed, helpless, weak and wished it had not happened that way.

*“Actually, my whole being was confused and I became totally weak but I acted bravely like a man. ….. then came back home.”* P 2012, a 30-year-old father of 4.

#### Burial

Participants reported that when the death was confirmed, the father of the deceased informed the family members and friends. Burial arrangement including transportation of the body, if death had occurred at a health facility was initiated. Most times, burial was conducted the following day or deferred by a day or two depending on the availability of the requirements, church ministers and mother’s relatives. Male participants reported that the wife’s relatives needed to view the body and understand the cause of death. Burial was arranged in a similar way as for a deceased adult but different from a miscarried baby. From the day death was confirmed, close relatives and friends converged at the bereaved home, they buried and stayed around a bonfire until the 3rd or 4th day for a baby girl or boy respectively. On the last day, ash was collected and disposed of by an elderly female relative. For young couples, the deceased was buried at the home of an elderly relative. Participants preferred a burial together with funeral prayers. This is because they believed prayers is the only way God can receive and keep the soul.

Participants reported that during this whole process, they were told not to cry. This was easy when many people were around but participants went back to grieving when they were left alone.

*“…………., people only came here in the evening, set fire and sat around it. In the morning the following day, they removed the baby’s body, took and buried it at the old Mama's place there led by a catechist”.* M9018, a 34-year-old single mother of 7.

#### Double loss – lost baby and financial resources

Most of the men felt in addition to losing a baby, they also lost resources. Most men narrated that they incurred expenses on medical care during pregnancy, childbirth and burial. This drained them economically and many had accumulated debts as one of them explained:

*“After the loss, I wasn’t at peace because I kept paying debts yet the baby was not there.” *P 2010, a 30-year-old father of 2.

#### Losing a baby is associated with multiple stressful roles

Male participants reported they were stretched by many activities that needed to be done at the same time following a perinatal death. Immediately after confirming the demise of their babies, male partners needed to transport the remains back home if death occurred in the hospital. Burial arrangements were disturbing because death is always abrupt and unexpected. Finances and food were often needed for burial and feeding mourners. They attended to the wives in the hospital and continuously supported them. Sometimes when death occurred and the lady was not officially married, her family members demanded marriage or compensation for the deceased baby before burial. These roles were reported as challenging because a father needed to attend to them in addition to mourning for the loss.

*“…. you know it's not easy to be a man. You have all the burden on your shoulders. Firstly, you need to comfort the wife so that she doesn't have many thoughts, then on the other side, you are also mourning. I also worried a lot about how the deceased and the mother would get back home.”* P 2001, a 25-year-old father whose baby died from a referral hospital.

#### Men prioritized their partner’s health

Most of the men who were interviewed reported that they were so worried about the recovery of their partners. This corresponds to a saying in *Lango* that “when the mother stone remains, you can still get new little ones”. They supported their wives to get medical treatment after childbirth. For these men, burying of the deceased baby was done in their absence. Men prioritized the recovery of their partners because that would help to get other children in future.

*“…….., I didn't mourn much about the children and I thought much about their mother. She was just operated on, she was unconscious! I only thought and worried that it was better to lose the children when their mother is alive.”* P 2014, a 44-year-old father of deceased twins.

#### Aggravating factors

There were several factors that intensified the grief of the perinatal death.

#### Health care provider reaction

Participants expressed mixed reactions regarding the behaviour of health care providers following a perinatal death. Most parents who had perinatal deaths from the health facilities felt unsupported by the health care providers. The nurses or midwives never talked to or consoled them, moreover, parents of the deceased babies felt the urgent need for support and knowledge on the cause of the death. Some participants felt the health care providers either did not know what to say or were too busy with their duties. Confirmation of death was not even made to some women and they discovered it on their own. Health care providers only came to administer treatment to them. Other participants, felt consoled and comforted by health care providers. They were encouraged to take heart because such things happen and they were neither the first nor the last. The mixed reactions could be because health care providers experienced many perinatal deaths routinely amidst their heavy workload. Therefore, they could be struggling to support the bereaved parents.

*“Oo the nurse—what they told me was that this thing has already happened. Now that your baby has passed on, you take it home and bury it. There is nothing much that we are going to tell you.”* P 2002, a 49-year-old father of 9.

#### Blame and guilt

Participants blamed the health care providers and the health system for the perinatal deaths. They described the delay and neglect by health care providers. Most times a health care provider delayed to attend or refer a pregnant mother in case they could not manage. In addition, participants stated that the absence of an ambulance coupled with bad roads contributed to death because they could not reach the referral health facilities in time. A male participant reported that mishandling their baby during birth resulted in the fracture of both limbs and an un-tied cord.

*“What I thought was not done, was at xx health centre here. If they had been fast enough…It would not have happened like that. They made me take long at xx here because I went on Sunday and then I was transferred to another facility on Thursday.”* M 9004, a 27-year-old mother of 2.

Some participants regretted and blamed themselves for lack of money and transport, delay to seek health care services, and childbirth at home. Women particularly blamed their partners and in-laws for not taking them to health facilities at the onset of labour, home delivery, and failure to buy drugs needed during pregnancy. Two women felt guilty for not delivering at the referral hospital where they could have had immediate surgical and neonatal care. One mother suspected that the problem with her womb to have caused the death. A male participant regretted taking a mother on a bicycle to the health facility over 3 km after the water broke. Some participants who had learned from the previous perinatal deaths sought health care early and had live babies at the time of the interview.

*“Lack of transport led to the death of that baby because it didn’t come out dead; It came out when it was still alive and got stuck halfway until its death. I regret that if I had been taken to the health centre that very night when the labour pain had just started, perhaps it couldn’t have happened that way.”* M 9006, a 35-year-old mother of 2.

Women were commonly blamed by their partners and in-laws for the perinatal deaths. One woman noted that “… it is more painful if you are accused by in-laws. Some kept worrying because the partners were being told to divorce them. These kept triggering the past pain and made them mourn for long. This could be because family members were searching for the cause of the perinatal deaths.

*“What a hard life! Sometimes your mind gets off because when you hear people's words, telling even your husband to just leave you and get another wife." *M 9013, a 24-year-old mother of 3.

### Sub-theme 2: delayed reaction to the perinatal loss

Participants reported experiencing reminders about the perinatal deaths. Marital challenges, the sight of other babies and subsequent pregnancy losses triggered the painful memory. When reminded, most parents noted that they did not have much pain compared to when the incident had just happened. This could mean that grief had reduced and the emotional reaction was not elicited. Some of them who did not have babies after the loss had thoughts and worries.

#### Marital challenges

Female participants noted that the presence of challenges in their marriages increased worries, pain and they would wish no child death had happened. On the other hand, women who reported no marital conflict claimed they did not have such worries.

*“. . . . . Violence is too much . . . . . At times, he says something that connects to the deceased baby. It annoys because it will be touching you who is wounded.” *M 9017, a 39-year-old mother of 10.

#### Sight of similar age children

One-third of the participants stated that they occasionally got reminded by the sight of babies born during the same period they experienced perinatal death. Most times, the mothers of the living children stay in the same area as them.

*“Why I think of it a lot is because there is – a child staying here, I went with her to the health centre one day, it can make me remember. Sometimes when I see that child, I remember then my mind would also let it go.”* M 9004, a 27-year-old mother of 2.

#### Consecutive pregnancy losses

Men whose partners experienced a perinatal loss in the subsequent pregnancy wished they had not lost the previous pregnancy. A male participant reported that his wife got four miscarriages within the first to second trimester after a previous early neonatal death. This was painful to him because it puts his wife in a worrying state.

*“After that baby, she kept having miscarriages, some would be three months old and others four months old. It happens, some at around two months, that was painful for me.” *P 2005, a 24-year-old father of 1.

### Theme 2: suggestions for support after a perinatal loss

#### Medical treatment of the deceased’ mother

Participants in the study made recommendations for medical treatment after a perinatal loss. They reported that after perinatal death, women experience painful complications postpartum which may need medical treatment. Participants commonly sought postpartum health care individually mainly from drug shops in their vicinity. Participants only went back to the health care facilities when they had serious complications.

*“…the most important assistance should be medical assistance given to a mother who has lost a baby.” P2004, a 27-year-old father of 2.*

#### Comfort and disclose the cause of a perinatal death

Participants reported the need for health care providers to comfort and console the parents after a perinatal loss. Comfort and consolation are required especially when a perinatal loss has just been confirmed in a health facility. This is likely to foster acknowledgement and a sign of care by health care providers. In addition, disclosure of the likely cause of perinatal death was required so that parents can prevent the re-occurrence of such losses. Parents also expressed a need for death certificates for a perinatal death as given for deceased adults. A male participant desired the need for health care facilities to assist with the transport of the deceased baby home.

*“. . . . for the nurses to assist the mothers who have lost their babies, they must be close to them to console them. If they investigate and find the real cause of that problem, they must advise so that such things don't repeat” *M 9014, 26-year-old-mother of an early newborn death.

#### Family and community support

Participants reported the importance of the family and community members’ support. They encouraged people to be close and console parents after perinatal death. Emphasis was put on appropriate ways of talking and advising a parent. They appreciate it if spoken to calmly and respectfully, for a bereaved parent to understand the advice. They desire continuity of emotional support even after the burial of the deceased baby. One participant appreciated and suggested women as great supporters owing to their ways of communication, closeness and patience. Some women felt the need to get over the grief and work hard to support their families. That would help the mind to slip away from the lost baby.

*“……….if you were to be left alone, you who has lost a dear one, you can think and lose your reasoning ability. That is why some people must stay with you for sometime…..”* M9018, a 35-year-old mother of 7.

## Discussion

This study was conducted to describe the lived experiences of parents following perinatal death in Lira district, Northern Uganda.

This study revealed that parents experienced pain and confusion following a perinatal death, consistent with previous studies [19, 34, 50–52]. The pain and confusion experienced could be a manifestation of the disparity between expectation of a baby who is alive and perinatal death [53]. Moreover, the occurrence of perinatal death is abrupt and unexpected, resulting in pain. The pain was expressed as heart-breaking, hurting and hard life. The expressions represent the processing of the pain of grief in Worden’s Task of Mourning [37]. The grief pain could be a normal reaction one has to go through to cope with the loss. Further, Worden affirms that the severity of grief pain is mediated by attachment with the deceased [37]. Parents are normally closely attached to the unborn because it is a social fulfilment for women while for men, it is the outcome of investment and hope of the family.

Participants reported that the deceased babies were similarly accorded burial as adults. Burial may be a sign of acceptance of the reality of the loss by family members as seen in Worden’s Task II of Mourning [37]. Communal burial is a common practice in the study area often enforced by *Lango* saying that “*Too obedo alea*” (Death alternates between people). This makes people stand with the bereaved family in anticipation of similar support when they lose a relative. Hence enforcing togetherness during the hard times. Public mourning and support during burial could be showing love and sympathy for the bereaved parents and a deceased baby. The adherence to cultural procedures may be to prevent bad omen or spiritual consequences on the parents and to promote coping. Preventing open mourning on the contrary encourages independent and silent grieving believed to be associated with later distress. Unique to this setting, telling parents to “stop crying, take heart and be strong” was accepted as a comforting message during the burial. The burial process in this setting was different from those reported in previous studies where burial was dependent on religion, and age, or was done in private [16, 54]. The difference could be attributed to the communal way of living among the Langi.

Men reported that they lost financial resources in addition to losing their newly born babies. This burden could be because in the study setting, health care services in government-aided facilities are free but because of frequent stock out of medical supplies and drugs, patients have to provide them. This is coupled with the low socioeconomic status of the participants in the study setting. The northern region of Uganda has been reported to have the highest proportion of chronically poor people (21.6%) compared to 4.9 and 0.5% in the Western and Central regions, respectively [55]. Unique to this study setting, people attach value and respect to the deceased body. Therefore, the cost of a decent burial according to the family’s social standing is the sole responsibility of the family members of the deceased. The presence of a big crowd during burial is more of an emotional but not a guarantee of financial support. Therefore, losing resources during health care and burial is likely to aggravate the emotional reactions. Financial strain has also been reported in the previous study to aggravate grief [17].

Findings showed that men had multiple roles including attending to the emotional needs of their spouses, transporting the deceased body back home and providing a decent burial. This could be because, in the study setting, males are brought up as heads of families and therefore culturally tasked with the responsibilities of the family. The stressing roles among men in this study is therefore uniquely associated with social-cultural roles and expectations. Previous studies from other contexts reported men as supporters of women [21, 56–58] even when they needed to grieve for the loss on their own. Furthermore, men are fathers and heads of families as such, they are involved in decision making and take on practical tasks about their loss [15, 21, 50, 56–59]. This implies that men who experience a perinatal death need to be supported in their roles.

There were mixed reactions regarding the conduct of health care providers following a perinatal death. Some health care providers were insensitive while others were supportive toward the participants. Although the experiences of the healthcare providers in this study setting are unknown; bereavement care is not addressed in the health care guidelines. Therefore, the care of the bereaved parents could be a personal initiative. Moreover, parents want health care providers to take time and sit with them, face to face and be present in their sadness [50]. This is important for the parents to receive emotional comfort and feel that their loss is acknowledged. This finding is consistent with previous studies where health care providers were insensitive and inconsiderate [16, 25, 60], partly because they distanced themselves from parents and focused on tasks as coping strategies [20]. Therefore, health care providers in the region need to provide culturally-sensitive support to parents who experience perinatal death.

Grief reactions among the participants were aggravated by blame and guilt. Blaming women undermines the heavyweight of the personal loss faced by a mother. Women in previous studies were also blamed for contributing to perinatal deaths [19, 52, 54]. Participants on the other hand blamed themselves and felt guilty for the loss. These regrets have been expressed in previous studies [17, 19, 21, 61]. Other than that, participants regretted the delay and neglect of the health care providers that contributed to the loss of their babies. Studies from other contexts reported similar actions of health care providers. Some parents felt health care providers were incompetent or not available when they were most needed similar to what has been documented by other studies [17, 21, 52]. Blame could be in an attempt to search for the cause of the death. As cited earlier, participants revisit the events in the process of trying to recognize the reasons for the baby’s death [61]. As recognized by Worden in his Tasks of Mourning [37], the experience of preventable death is associated with guilt or blame as a mediator in the process of mourning which is likely to affect the coping of the bereaved. Therefore, this calls for health care providers to acknowledge and assure the parents of their ability to bear more children after the loss. Parents also need to be educated on the causes of deaths to minimize blames.

At a later time, participants were reminded of their lost babies when they saw babies of the same age. This could be because seeing the babies psychologically reminds the parents of the sad events and the permanent gap created by the perinatal death in their family. The sight of babies was reported as a trigger of grief in other contexts [52, 62]. Other participants also felt marital conflicts triggered their past painful memory. Women who have undergone a perinatal loss are so sensitive to the words and actions of their family members. Verbal exchanges or actions that do not comfort them are negatively perceived as affecting their grief. Marital challenges have been reported as part of perinatal grief [17]. Subsequent pregnancy losses elicited the grief reaction. This implies the need for postpartum care to prevent the re-occurrence of death. Women should be given continuous emotional support and encouragement to facilitate recovery from the painful experience.

The participants in the study recommended health care attention for postpartum complications after the loss. Other suggestions included health care providers’ comfort, consolation and disclosure of the cause of death as well as family and community members’ presence to share in their grief. Taken together, the experiences and suggestions of parents in this study, reflect a need to strengthen formal and informal support systems for those who experience a perinatal loss. A study conducted in Eastern Uganda revealed a general lack of formal systems to care for families that faced a stillbirth [16]. At the health system level, there is a need to train health care providers in bereavement counselling and support. Further, availability of personnel and adequate medical supplies are needed to attend to the needs of mothers during the grieving period and for subsequent pregnancies to avoid the re-occurrence of such losses. Women also expressed the need for assurance that health care providers did their best to prevent death and that such incidents would not re-occur in future. At the community level, health care providers should educate families and communities on the causes of perinatal deaths to reduce blame, stigmatization and threats of divorce for women who experience perinatal losses.

### Strengths and limitations

This study is unique in the sense that it is one of the few studies that have described the experiences of women and men who had a perinatal death in a rural setting of Northern Uganda. Inclusion of men and women in our study provided room for triangulation which improved trustworthiness of study findings. The participants were interviewed individually and not as a couple which allowed each participant to speak freely, irrespective of what the partner thinks. Interviews were conducted by researchers familiar with the language and culture in the study setting. Also, the lead author had a 2-year extended stay in the area during data collection and as part of the Survival Plus project which further facilitated an in-depth understanding of the context surrounding perinatal deaths and informed interpretation of study findings. The study had male and female research assistants. The lead interviewers were the same sex as the participants which facilitated study participants to speak freely, thus improving the validity of findings. The authors feel the interviews were conducted until saturation both for male and female participants. However, our findings should be interpreted in view of the following limitations: 1) Husband-wife pairs were not included in this study, this limits generalization to couples’ experience after a perinatal death. 2) Other family members were not interviewed which would have further enriched understanding of experiences of loss in a family setting as a support unit. 3) Translation of transcripts from *Lango* to English could have diluted some verbal expression of the participants’ experiences. However, checks of transcripts against audios by research team members found that the transcripts were in general accurate which is re-assuring that the limitation of diluted expressions due to translation could have been very minimal. 4) The study was conducted in one rural region of Uganda and thus applicability of findings to other regions with varying culture and socio-economic status may be limited. However, the fact that some of the findings in our study on the experience of pain, confusion, grief and marital challenges by parents following a perinatal death are similar to what has been documented in other settings [19, 52] is reassuring that our findings may have wider relevancy beyond the study setting.

## Conclusion

Parents who experienced a perinatal death were affected emotionally, physically, socially and economically. Unsupportive behaviour of health care providers, financial constraints and feelings of guilt aggravated the grief reaction. Postnatal care, acknowledgment and emotional support of the grieving parents are suggested after a perinatal loss. There is need to train health care providers in bereavement counselling to enable them better support the grieving parents. In addition to family and community members, health care providers need to provide emotional support and postnatal care to parents who experience a perinatal death so that subsequent pregnancy losses are avoided. Bereaved men uniquely need support with the multiple roles so that they are able to mourn for their loss. There is need for further studies to explore the perception of close family members and health care providers in the region towards perinatal loss.

## Data Availability

The datasets generated and analysed during the study are not publicly available as the data cannot be anonymised adequately for public sharing. The data may be available from the corresponding author on a reasonable request.

## Abbreviations

LMIC: Low- and middle-income countries
IDI: In-depth interviews
MA: Masters of Arts in Applied African Linguistics
